# Signaling through the Primary Cilium

**DOI:** 10.3389/fcell.2018.00008

**Published:** 2018-02-08

**Authors:** Gabrielle Wheway, Liliya Nazlamova, John T. Hancock

**Affiliations:** Department of Applied Science, Faculty of Health and Applied Sciences, Centre for Research in Biosciences, University of the West of England, Bristol, United Kingdom

**Keywords:** cell signaling, primary cilium, ciliopathies, Hedgehog, development, developmental disorders

## Abstract

The presence of single, non-motile “primary” cilia on the surface of epithelial cells has been well described since the 1960s. However, for decades these organelles were believed to be vestigial, with no remaining function, having lost their motility. It wasn't until 2003, with the discovery that proteins responsible for transport along the primary cilium are essential for hedgehog signaling in mice, that the fundamental importance of primary cilia in signal transduction was realized. Little more than a decade later, it is now clear that the vast majority of signaling pathways in vertebrates function through the primary cilium. This has led to the adoption of the term “the cells's antenna” as a description for the primary cilium. Primary cilia are particularly important during development, playing fundamental roles in embryonic patterning and organogenesis, with a suite of inherited developmental disorders known as the “ciliopathies” resulting from mutations in genes encoding cilia proteins. This review summarizes our current understanding of the role of these fascinating organelles in a wide range of signaling pathways.

## An introduction to the primary cilium

The primary cilium is a long, thin organelle protruding from the apical surface of almost all cell types, most commonly from epithelial cells. This structure is formed when the cell is in G0 or G1 phase, and often during S/G2 phase (Plotnikova et al., 2009). The timing of cilium formation, “ciliogenesis,” is restricted to these stages of the cell cycle because the cilium is rooted at its base by the basal body, which is derived from the mother centriole of the centrosome (Nigg and Stearns, 2011). The centrosome has an essential function in nucleating the mitotic spindle during cell division, so prior to mitosis the cilium is resorbed to release the centrioles, and ciliogenesis commences again shortly after cytokinesis is complete (Basten and Giles, 2013). The cilium is a characteristic feature of post-mitotic epithelial cells and differentiated cells which have exited the cell cycle. Since Sorokin (1962) first described primary cilia on fibroblasts and smooth muscle cells, these organelles have been observed on almost every cell type of the human body.

**Figure 1:**
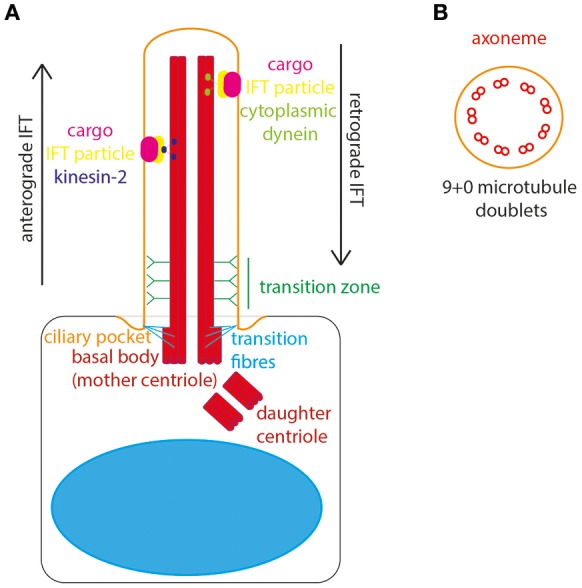
The structure of the primary cilium. **(A)** The primary cilium is formed on the apical surface of cells, from the basal body which is derived from the mother centriole. The daughter centriole stays associated, at roughly rightangles, surrounded by pericentriolar material. The basal body is located in a depression of membrane known as the ciliary pocket, and is connected to membrane here by transition fibers. The region where the central pair of microtubules in the basal body microtubule triplet grow to form the ciliary axoneme is called the transition zone. Here, Y-linkers connect the axoneme to the ciliary membrane. Protein and other cargos are transported from cilium base to tip by anterograde IFT particles and kinesin-2 motor. Protein and other cargos are transported from cilium tip to base by retrograde IFT particles and cytoplasmic dynein motor. **(B)** In cross-section the 9+0 formation of microtubule doublets can be seen in radial array, making up the ciliary axonome. It is formed of a ring of 9 pairs of post-translationally modified microtubules, with no central pair and no dynein arms.

The basal body is composed of a ring of 9 triplets of gamma tubulin and docks at the apical cell surface to define cell polarity and initiate ciliogenesis. From the apical surface of the basal body the main body of the cilium, the “axoneme,” emanates from the cell surface (Figure 1A). The axoneme is a microtubule structure of alpha and beta tubulin, post-translationally modified to stabilize it from depolymerisation. These microtubules form a radial array of 9 doublets. The absence of a central pair of microtubules distinguishes the primary cilia from motile cilia. The motile cilia has typically a “9+2” structure, with dynein arms moving against the central pair to initiate ciliary movement. Non-motile primary cilia lack this central pair and dynein arms, they have a “9+0” structure and therefore lack motility (Figure 1B). The entire axoneme is encased by membrane continuous with the plasma membrane of the cell.

The cilium is divided into subdomains. At the very base lies a small “ciliary pocket” where the membrane is depressed slightly (Molla-Herman et al., 2010). Within this pocket the basal body is located, with transition fibers connecting the basal body microtubules to the cilium membrane. The transition fibers are the site of vesicle docking, where vesicles carrying new cilium membrane lipids and transmembrane proteins are processed for entry in to the cilium (Reiter et al., 2012). The innermost and middle microtubules of the basal body triplet form the anchor for the doublet which grows to form the ciliary axoneme. The region of conversion from microtubule triplet in the basal body to microtubule doublet in the axoneme is known as the “transition zone.” In the transition zone Y-shaped links connect the axonemal microtubules to the cilium membrane, appearing as a “ciliary necklace” when viewed under freeze-fracture scanning electron microscopy. Collectively, the transition fibers and transition zone form the ciliary gate, where entry and exit of cilium proteins and lipids is controlled (Garcia-Gonzalo and Reiter, 2017; Figure 1A). In this way, the cilium is continuous with the main cell body, whilst remaining a distinct and discrete organelle with its own proteome (Gherman et al., 2006).

The transition fibers are also the site of docking of intraflagellar transport (IFT) particles, which carry protein and other cargos into the ciliary compartment. IFT is the process by which all proteins are transported into and along the ciliary compartment, as protein translation cannot occur here. Anterograde IFT transports proteins along the cilium from base to tip, catalyzed by cytoplasmic dynein 2/1b motor whereas retrograde IFT transports proteins from the tip to the base of the cilium, catalyzed by kinesin-2 motor protein (Ishikawa and Marshall, 2017). The importance of this process for normal cilium structure and function was uncovered by Pazour et al., who discovered that IFT88 mutations in *Chlamydomas* resulted in absence of flagella, and in mice resulted in cilia defects leading to polycystic kidney disease (Pazour et al., 2000). Thus, the first links were made between primary cilia and genetic disease. It is now known that defects in primary cilia are associated with a broad suite of inherited developmental and degenerative conditions affecting multiple organs and organ systems–the ciliopathies (Waters and Beales, 2011).

Of significance for this review, primary cilia are fundamentally important for normal cell signaling during development and homeostasis, resulting in the adoption of the term “cell's antenna” when referring to the primary cilium (Singla and Reiter, 2006). These signaling functions are carried out by the myriad of signaling molecules localized to the primary cilium. Transmembrane receptors embedded in the cilium membrane allow the cell to respond to various external stimuli, and regulatory proteins at the basal body, transition zone and distal regions of the primary cilium control the signaling cascades. A diverse array of signaling pathways have been linked to the cilium, including Hedgehog, Wnt, Notch, Hippo, GPCR, PDGF (and other RTKs including FGF), mTOR, and TGF-beta.

The signaling function for which the primary cilium is perhaps best known is Hedgehog signaling.

## Hedgehog signaling through the primary cilium

**Figure 2:**
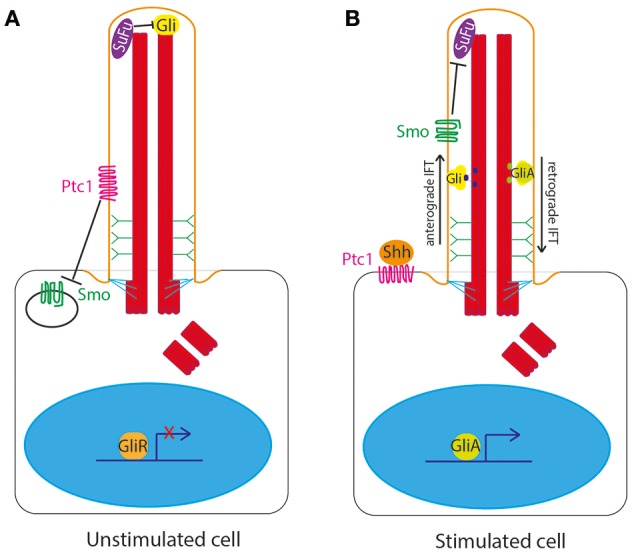
Hedgehog signaling at the primary cilium in vertebrates. **(A)** In the unstimulated state, Ptc1 sits in the cilium membrane and represses and excludes Smoothened (Smo) from the cilium. Gli transcription factors are sequestered and suppressed by Suppressor of Fused (SuFu) at the tip of the primary cilium. **(B)** In the stimulated state, upon binding of Shh to Ptc1, the repression of Smo by Ptc1 is relieved, allowing Smo to enter the cilium and Ptc1 to leave the cilium. This then allows Smo to repress SuFu, relieving repression of Gli at the tip of the cilium. Gli is thus freed to be post-translationally modified to form Gli activator form (GliA), which is transported out of the cilium to the nucleus to activate expression of downstream target genes.

The primary cilium is the central organelle for the transduction of the Hedgehog signaling pathway in vertebrates. The cilium membrane is the site of location of Ptc1, the 12 transmembrane domain receptor of Shh ligand. In the unstimulated state, Ptc1 sits in the cilium membrane and represses and excludes Smoothened (Smo) from the cilium. Gli transcription factors are sequestered and suppressed by Suppressor of Fused (SuFu) at the tip of the primary cilium in the unstimulated state (Haycraft et al., 2005; Zeng et al., 2010; Figure 2A).

Upon binding of Shh to Ptc1, the repression of Smo by Ptc1 is relieved, allowing Smo to enter the cilium and Ptc1 to leave the cilium (Corbit et al., 2005; Rohatgi et al., 2007). This then allows Smo to repress SuFu, relieving repression of Gli at the tip of the cilium. Gli is thus freed to be post-translationally modified to form Gli activator form (GliA), which is transported out of the cilium to the nucleus to activate expression of downstream target genes (Figure 2B).

Movement of Hh signaling intermediates into and out of the cilium is facilitated by IFT proteins and IFT motor proteins. It was the study of IFT mutant mice which offered the first insights into the role of the primary cilium in the Hedgehog pathway, with these mice displaying classic Shh phenotypes. These IFT proteins were subsequently shown to be required for Hh signaling downstream of Ptc1 and upstream of Hh signaling gene targets (Huangfu et al., 2003). Loss of IFT proteins leads to downregulation of Ptc-1 expression (Beales et al., 2007) and accumulation of Gli2 and Gli3 at the cilium tip (Qin et al., 2011). This is under the control of KIF7, anterograde IFT motor protein, which regulates the length of the axoneme by control of growth of microtubules at the cilium tip (Pedersen and Akhmanova, 2014). Recent work suggests that loss of IFT80 prevents Smo localization to the cilium ciliary, inhibiting canonical Hh signaling, but increases Smo and Gαi binding, leading to increase non-canonical Hh-Gαi-RhoA-stress fiber signaling in differentiating osteoblasts (Yuan et al., 2016).

The role of primary cilia in transduction of Hedgehog signaling is complex and context-dependent, and cilia can act as both positive and negative regulators of the Hedgehog signaling pathway. Defects in cilia and IFT result in loss of function Hh phenotypes in the neural tube (where Gli activators normally play a major role) and gain of function Hh phenotypes in the limb (where Gli3 repressor normally plays a major role) (Haycraft et al., 2005; Huangfu and Anderson, 2005). Foxj1, a transcription factor which plays a fundamental role in the formation of motile cilia, has been shown to function in an antagonistic manner toward the Gli transcription factors to pattern the developing neural tube, and cilia are necessary for this function of Foxj1 in the Shh pathway (Cruz et al., 2010).

*Rpgrip1l*, mouse homolog of a human ciliopathy protein, which has been shown to be essential for Hh responsiveness, and mutant mice have left-right patterning defects, neural tube defects and limb patterning defects (Vierkotten et al., 2007). The involvement of cilia in Hh signal transduction helps to explain the common Hh-type phenotypes seen in many ciliopathies, such as midline defects (Chiang et al., 1996), neural tube defects (Echelard et al., 1993) polydactyly (Hui and Joyner, 1993; Riddle et al., 1993), lung hypoplasia (Warburton et al., 2000), and coloboma (Schimmenti et al., 2003). All of these features are seen in Meckel-Gruber syndrome, the most severe ciliopathy (Wheway et al., 2014; Hartill et al., 2017; Figure **7**, Table 1). Polydactyly is a feature of several other severe ciliopathies, including Joubert syndrome, Bardet-Biedl syndrome and Oro-facial digital syndrome, and neural tube defects are a feature of Meckel-Gruber and Joubert syndromes (Waters and Beales, 2011).

**Table 1:** Ciliopathy phenotypes, the ciliopathies which develop these phenotypes, and the signaling pathway underlying the phenotype.

**Ciliopathy phenotype**	**Ciliopathy**	**Underlying signaling defect resulting from cilium loss or dysfunction**
Polydactyly	BBS, JATD, JBTS, MKS, OFD	Shh
Sensorineural deafness	ALMS, USH	Wnt PCP
Occipital encephalocele and other neural tube defects/midline defects	MKS, OFD	Shh, Wnt PCP, Notch
Situs defects	MKS	Notch, LR
Kidney fibrosis and inflammation	NPHP, SLS	Hippo
Obesity/endocrine disorders	ALMS, BBS	GPCR (gonadotropin hormone receptor)
Complex neurodevelopmental defects including cerebellar vermis hypoplasia, ataxia, and psychomotor delay	JBTS	GPCR (ARL13B), PDGFR (INPP5E), Shh, Wnt
Cystic kidney disease	BBS, JBTS, MKS, OFD, PKD	mTOR, canonical Wnt
Retinal degeneration	ALMS, BBS, JATD, JBTS, LCA, RP, SLS, USH	GPCR (rhodopsin/opsin)

*ALMS, Alström syndrome; BBS, Bardet-Biedl syndrome; JATD, Jeune asphyxiating thoracic dystrophy; JBTS, Joubert syndrome; LCA, Leber congenital amaurosis; MKS, Meckel-Gruber syndrome; OFD, Oro-facial-digital syndrome; PKD, Polycystic kidney disease; RP, retinitis pigmentosa; SLS, Senior-Løken syndrome; USH, Usher syndrome*.

There is increasing evidence that cilia may also play dual roles in promoting or inhibiting progression of cancer, where the Hedgehog pathway is commonly dysregulated. In several types of cancer, where Hedgehog signaling is upregulated, tumor cells are reported to significantly lack cilia compared with cells from surrounding normal tissue (Moser et al., 2009; Seeley et al., 2009; Yuan et al., 2010; Kim et al., 2011). This can either have a positive effect, in tumors driven by activation of Smoothened (Smo) or negative effect in tumors driven by activation of Gli2, a downstream transcription factor (Han et al., 2009; Wong et al., 2009).

Conversely, the Hh pathway may also regulate primary ciliogenesis and maintenance in a feedback loop. A genome-wide RNA interference (RNAi) screen revealed a number of Hh pathway genes which, when knocked down, led to cilia disassembly, including a protein called Stk11, also known as Lkb1, which also regulates the Wnt signaling pathway (Jacob et al., 2011).

It is well established that cilia play a central role in Shh signal transduction, with functional cilia and IFT essential for normal Shh signaling. However, the exact role of cilia in the Shh pathway is context-dependent, with different roles in tissues where Gli activators normally play a major role compared to tissues where Gli3 repressors normally play a major role. The contribution of Shh defects to various ciliopathy phenotypes such as polydactyly is well characterized, but the extent to which cilia play a role in defective Shh signaling in cancers requires considerably more study.

## Wnt signaling through the primary cilium

**Figure 3:**
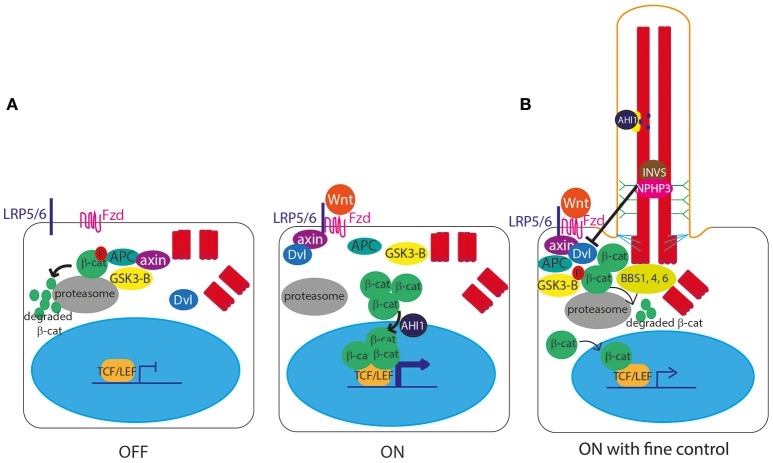
Canonical Wnt signaling at the primary cilium. **(A)** In the unstimulated “off state,” the Axin/APC/GSK3-β “destruction complex” targets β-catenin to the proteasome for degradation, preventing β-catenin from entering the nucleus to activate gene expression. In the stimulated “on” state, Wnt ligands bind to a membrane-bound Frizzled (Fzd) receptor, which then binds LRP5/6, allowing it to recruit Axin. With Axin sequestered by LRP5/6, the Axin/APC/GSK3-β “destruction complex” can no longer degrade β-catenin, leaving it free to enter the nucleus, aided by AHI1, to interact with TCF and LEF transcription factors to activate transcription of Wnt target genes under TCF/LEF promoters. The Wnt signal is transduced via Disheveled (Dvl), which is recruited to the membrane and binds Axin upon stimulation. **(B)** The primary cilium controls the level of expression of Wnt target genes, via controlled degradation of Dvl by cilia proteins INVS and NPHP3, and by sequestering AHI1 at the cilium so it cannot aid translocation of β-catenin into the nucleus.

The role of cilia in canonical Wnt signal transduction activation (Clevers and Nusse, 2012; Figure 3A) remains somewhat controversial, with some publications showing data supporting the hypothesis of a link between cilia and Wnt signaling, and others disputing this. IFT mutant zebrafish lacking cilia retain normal canonical and non-canonical Wnt signaling (Huang and Schier, 2009) and IFT mutant mice show normal expression of Wnt targets, normal activation of a transgenic Wnt reporter, and normal response to Wnt ligands in culture (Ocbina et al., 2009). Similarly, no defects in canonical Wnt signaling were found in mice lacking Inversin (Invs), the mouse homolog of a protein encoded by *NPHP2*, which is mutated in nephronophthisis, a degenerative renal ciliopathy (Sugiyama et al., 2011; Figure **7**, Table 1).

However, an earlier study suggested that Invs inhibits canonical Wnt signaling by targeting cytoplasmic Disheveled for degradation (Simons et al., 2005; Figure 3B). Another study showed that NPHP3, another cilia protein mutated in nephronophthisis, is involved in this pathway (Bergmann et al., 2008; Figure 3B). Further to this, there is significant published data suggesting that primary cilia play extremely important functions in attenuation of the canonical Wnt signaling pathway, with several cell and animal studies showing that defects in cilia lead to massive over-activation of Wnt signaling, including in mouse models of Meckel-Gruber syndrome (Lin et al., 2003; Cano et al., 2004; Abdelhamed et al., 2013; Wheway et al., 2013).

Conversely, *Ahi1* mutant mice (the homolog of *AHI1* which encodes Jouberin, a cilia protein) show a loss of basal canonical Wnt signaling activity leading to cystic kidney disease (Lancaster et al., 2009). *AHI1* mutations in humans cause Joubert syndrome, a severe multi-organ ciliopathy which sometimes presents with polycystic kidneys (Ferland et al., 2004; Figure **7**, Table 1). This control of Wnt signaling is achieved by sequestration of Jouberin in the primary cilium, away from the nucleus. This limits entry of ß-catenin into the nucleus, restricting but not totally inhibiting the activation of downstream Wnt target genes (Lancaster et al., 2011; Figure 3B).

It is thought that several proteins associated with Bardet-Biedl syndrome, a severe multi-organ ciliopathy (Figure **7**, Table 1), play a role in this Wnt signal regulation via targeted proteasomal degradation of Wnt effectors (Gerdes et al., 2007; Wiens et al., 2010; Figure 3B).

**Figure 4:**
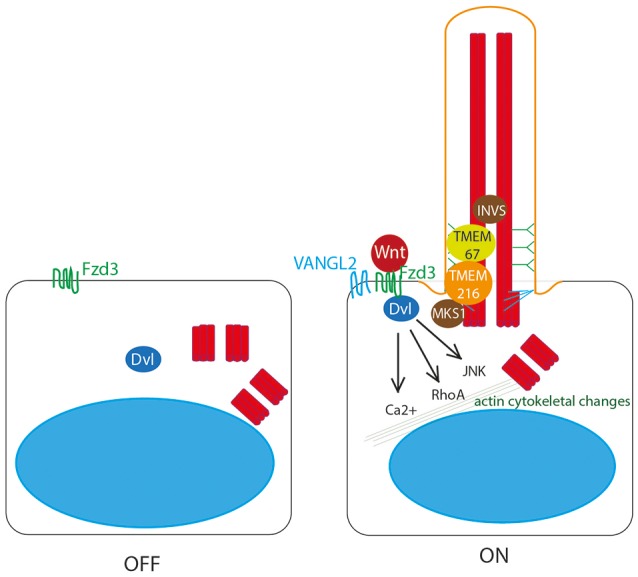
Non-canonical Wnt signaling at the primary cilium. Non-canonical Wnt ligands bind to Frizzled 3 (Fzd3) receptor, which triggers asymmetric localisation of Vangl2 in the cell. This pathway acts through Dvl to activate RhoA, and the JNK pathway, to stimulate Ca^2+^ release to stimulate remodeling of the actin cytoskeleton. This is dependent upon correct definition of cell polarity by basal body migration to the apical cell surface. This migration is regulated by Dvl, by transition zone proteins meckelin (TMEM67) and TMEM216 and by basal body protein MKS1. Inversin also plays a role.

Whilst the role of cilia in canonical Wnt signal transduction is disputed, it is well accepted that normal ciliogenesis is essential for the planar cell polarity (PCP) non-canonical Wnt signaling pathway (Gomez-Orte et al., 2013; Figure 4). This process depends on cell polarity being correctly established, which is dependent upon migration of the basal body to the apical cell surface to define apicobasal polarity (Jones et al., 2008). This apical positioning of the basal body in the establishment of PCP is a highly conserved feature across evolution, and centriolar positioning is considered a fundamental functional readout of PCP (Carvajal-Gonzalez et al., 2016). Thus, defects in proteins regulating initiation of ciliogenesis and basal body migration lead to complex PCP defects, manifesting in gastrulation defects, neural tube defects, and inner ear defects as the cochlea hair cells do not grow stereocilia in the correct orientation. Defects in cilia proteins can thus lead to inherited forms of congenital deafness alongside retinitis pigmentosa in a condition known as Usher syndrome (Sorusch et al., 2014; Figure **7**, Table 1).

Dvl is essential for this process of basal body docking, ciliogenesis and PCP (Wallingford et al., 2000; Park et al., 2008) as are transition zone proteins meckelin (TMEM67) and TMEM216, and basal body protein MKS1 (Figure 4), which are mutated in Joubert syndrome and Meckel-Gruber syndrome, the most severe ciliopathies (Dawe et al., 2007, 2009; Valente et al., 2010; Adams et al., 2012; Figure **7**, Table 1). BBS10 and 12, chaperonin proteins, are thought to also play a role in this process (Seo et al., 2010). Concurrent to its role in restricting canonical Wnt signaling, Inversin also enhances non-canonical Wnt, and in this way is thought to control the switch between canonical and non-canonical Wnt signaling in *Xenopus* (Simons et al., 2005). Loss of any of these proteins leads to significant planar cell polarity defects.

Whilst the role of cilia in regulating canonical Wnt signaling remains controversial, and requires further investigation, the importance of the basal body in establishing non-canonical Wnt PCP is well established. Migration of the basal body to the apical cell surface is essential for PCP, and is coordinated by a number of basal body and transition zone proteins which are mutated in ciliopathies.

## Notch signaling through the primary cilium

**Figure 5:**
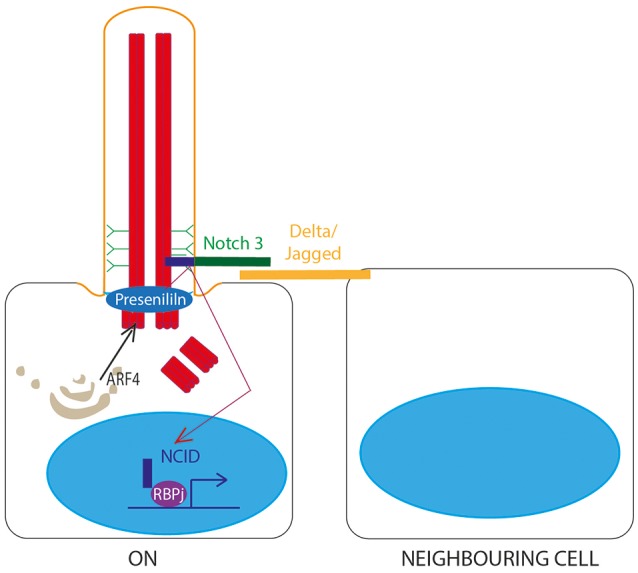
Notch signaling through the primary cilium. A Notch receptor binds to a membrane-bound Delta or Jagged ligand on an adjacent cell, stimulating proteolytic cleavage of the Notch intracellular domain (NCID) by Presenilin, allowing NCID to translocate to the nucleus where it can activate downstream target genes in association with RBPj DNA binding protein. This is dependent on correct localisation of Presenilin to the basal body, controlled by ARF4 exocytosis.

A role for the primary cilium in Notch signaling (Guruharsha et al., 2012; Figure 5) was first identified in 2011 when knockdown of IFT proteins in keratinocytes and developing embryos was shown to lead to dysregulated Notch signaling, increased proliferation and defects in differentiation. Notch3 receptor and Notch-processing enzymes colocalise with cilia in wild-type epidermal cells, and loss of cilia *in vivo* leads to significant Notch defects and failures in differentiation of basal cells to the spinous cell fate in developing epidermis (Ezratty et al., 2011). Subsequent work showed that layers of epidermis with the most primary cilia had the highest levels of Notch signaling, and in these cells, Presenlin, a key regulator of Notch signaling, was localized to the basal body, controlled by ARF4 exocytosis (Figure 5). This suggests that the primary cilium regulates Notch signaling by regulating spatial localization of Notch signaling intermediates during epidermal differentiation (Ezratty et al., 2016).

Conversely, loss of primary cilia in corneal epithelia leads to diminished Notch activation, with reduced levels of nuclear Notch1 intracellular domain (N1ICD), leading to reduced cell proliferation (Grisanti et al., 2016).

As with many signaling pathways, the role of primary cilia in Notch signal transduction is highly context dependent. In the neuroepithelia of the developing neural tube, activation of Notch signaling leads to increased primary cilium length and accumulation of Smo in the primary cilium (Stasiulewicz et al., 2015). This encourages further expression of Shh, leading to more prolonged exposure of cells to higher levels of Shh, which specifies the ventral cell fate in the developing neural tube. Shh is secreted by the notochord, and the dorso-ventral patterning of the overlying neural tube is established depending on the level of exposure to Shh. Notch augments this responsiveness, via the primary cilium. Whilst incredibly important for normal development (many ciliopathies exhibit neural tube defect phenotypes), somatic cell mutation leading to misactivation of Shh/Notch signaling in the primary cilium of choroid plexus tumors has been shown to drive this particular type of cancer (Li et al., 2016).

Glycosylation of Notch also plays a role in regulation of cilia function at the embryonic node, where a mixed population of motile and non-motile cilia establishes leftward fluid flow to define the left-right (LR) asymmetry of the embryo. Glycosylation of Notch1 activates the signaling pathway which increases specification of non-motile primary cilia at the node. Loss of this process leads to situs defects as a result of disturbed fluid flow at the node (Boskovski et al., 2013; Tavares et al., 2017).

A more recent discovery, it is now becoming apparent that Notch signals through the primary cilium in specific cell types during development. In keratinocytes, corneal epithelia and neuroepithelia at least, cilia are required to regulate Notch signaling to ensure proper control of cell differentiation and proliferation. Future studies of other cell types may reveal further roles for the cilium in Notch transduction.

## Hippo signaling through the primary cilium

**Figure 6:**
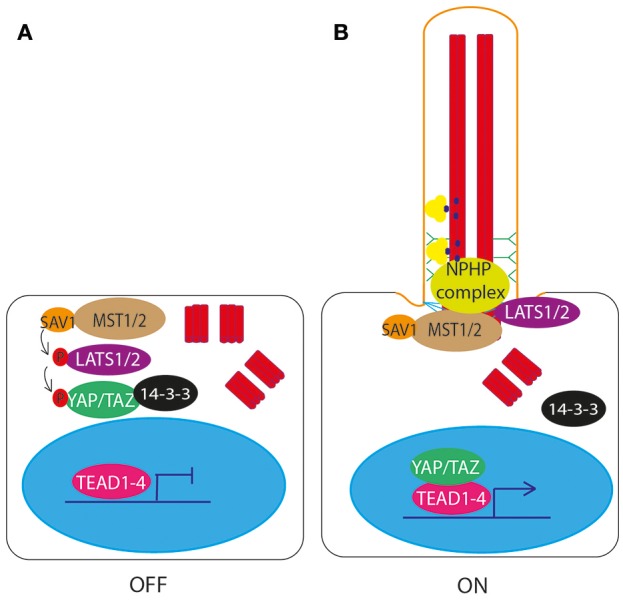
Hippo signaling through the primary cilium. **(A)** In the unstimulated “off” state, MST1/2 phopsphorylates LATS1/2, resulting in the downstream phosphorylation and inactivation of YAP/TAZ. Phosphorylation of YAP/TAZ causes it to bind 14-3-3 and be retained in the cytoplasm, preventing it from complexing with TEAD1-4 to activate transcription of target genes. **(B)** In the ciliated “on” state, NPHP complex proteins bind MST1/2 at the basal body, and LATS1/2, preventing phosphorylation and activation of YAP/TAZ. YAP/TAZ is then free to enter the nucleus and complex with TEAD1-4 to activate transcription of target genes.

One of the core components of Hippo signaling genes (Yu and Guan, 2013; Figure 6A), MST1/2 (Hippo), has recently been shown to localize to the basal body and be required for ciliogenesis (Figure 6B). Loss of MST1/2 or SAV1, which helps to activate MST1/2, results in impaired ciliogenesis. This is because MST1/2 is required for phosphorylation of Aurora kinase A (AURKA) to prevent it from complexing with HDAC6 to disassemble cilia. Further to this, MST1/2-SAV1 promotes ciliogenesis via association with the NPHP complex which regulates ciliary loading of cargoes into IFT transport machinery for transport into the cilium at the transition zone (Kim et al., 2014; Figure 6B).

**Figure 7:**
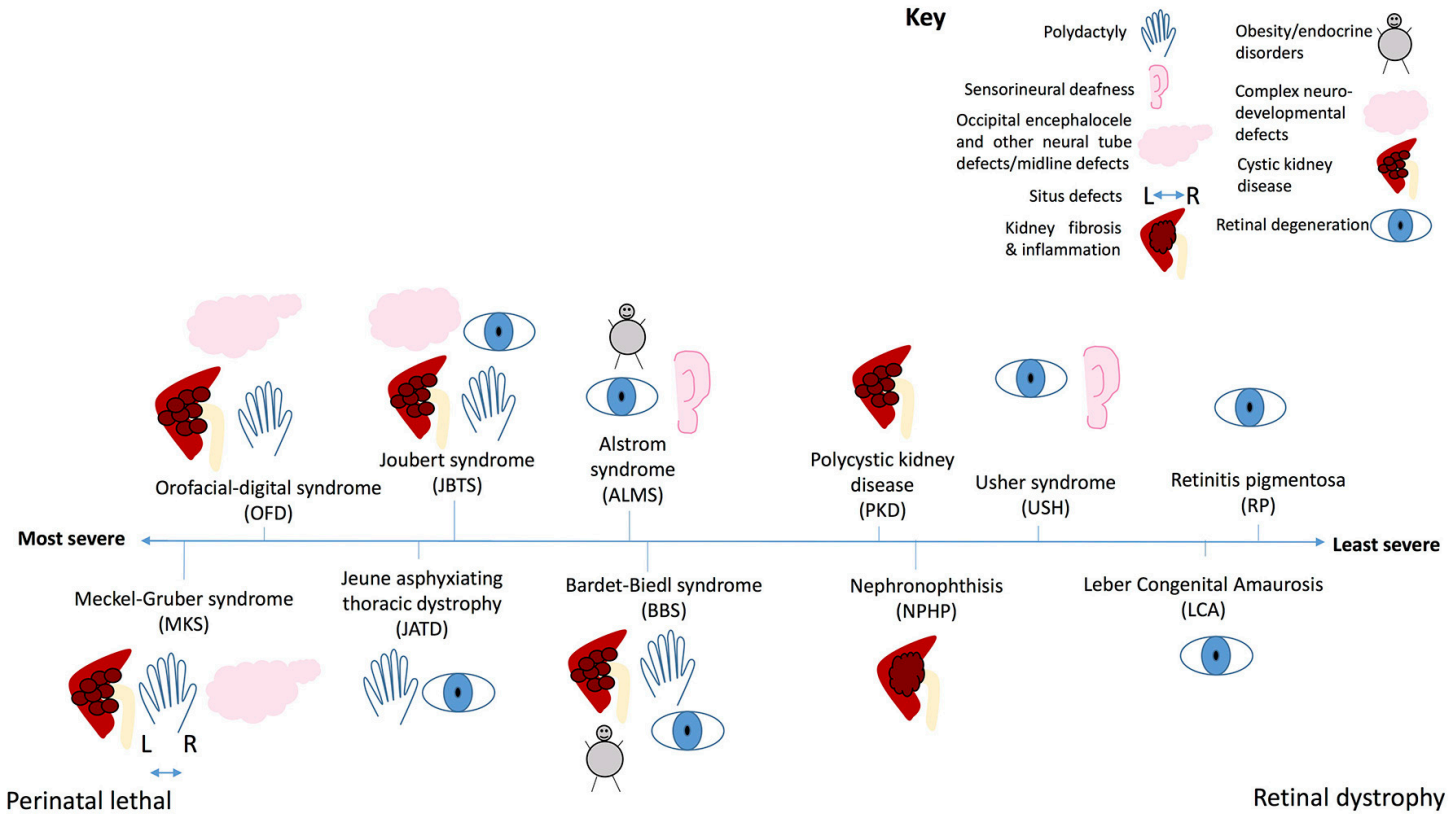
The ciliopathy spectrum. Schematic illustration of common features of ciliopathies, and severity of each ciliopathy along a spectrum from perinatal lethal to isolated retinal dystrophy. Key shows which phenotype is represented by each symbol.

The NPHP proteins are mutated in patients with nephronophthisis, a ciliopathy affecting the kidneys, characterized by fibrosis and corticomedullary cysts. NPHP4 regulates the Hippo pathway by binding to LATS1/2 and preventing it from phosphorylating YAP/TAZ, allowing YAP/TAZ to enter the nucleus and activate gene transcription (Habbig et al., 2011; Figure 6B). NPHP proteins NEK8 (NPHP9) and NPHP3 form a complex which activates YAP/TAZ (Frank et al., 2013, with NEK8 (NPHP9) stimulating nuclear translocation of YAP/TAZ, leading to downstream activation of gene targets (Habbig et al., 2012; Figure 6B). Loss of these proteins leads to altered Hippo signaling. It is thought that this dysregulation of Hippo signaling contributes to disease phenotype in patients with mutations in these genes (Figure 7, Table 1).

Other proteins known to be essential for ciliogenesis, such as EXOC5, have been shown to regulate the Hippo pathway. Loss of EXOC5 leads to loss of cilia, increased phosphorylation of MOB, the protein which regulates LATS1/2 (Lobo et al., 2017).

Perhaps the most recent discovery in the study of ciliary signaling, the Hippo pathway acts through proteins localized to the basal body of the cilium, where NPHP proteins regulate this pathway's activity through promoting phosphorylation and nuclear translocation of pathway activators.

## PDGFR signaling through the primary cilium

PDGFRα signaling is regulated through the primary cilium in quiescent fibroblasts, mesenchymal-derived cells, and PDGFR α localizes to the primary cilium during growth arrest to activate the MEK1/2-ERK1/2 and Akt pathways (Schneider et al., 2005). PDGFaa ligand binds PDGFR α in the primary cilium membrane to reorganize the cytoskeleton to drive directional cell migration of fibroblasts in wound healing, and fibroblasts from mutants with defective cilia show abnormal wound healing (Schneider et al., 2010). PDGFaa enhances Inversin-P-Akt localisation to the basal body, loss of Akt reduces cilia growth (Suizu et al., 2016).

Signaling through PDGFRs has been linked with resorption of cilia, a key step in cell cycle progression to allow the centrioles to participate in mitosis. Wild-type PDGFR beta and mutant PDGFR alpha trigger deciliation via PLC gamma and intracellular calcium release (Nielsen et al., 2015). It is thought that phosphatase and tensin homolog, PTEN, an antagonist of PI3K, contributes to this deciliation through regulation of phosphorylation of Disheveled (Shnitsar et al., 2015). Inositol polyphosphate 5-phosphatase (INPP5E), a protein which hydrolyses downstream products of PDGF signaling activation, also plays a role in this process. INPP5E mutation leads to increased ciliary PDGFRα signaling and premature disassembly of the cilium, followed by accelerated cell cycle entry. INPP5E is mutated in Joubert syndrome, a severe ciliopathy (Bielas et al., 2009; Jacoby et al., 2009; Figure 7, Table 1).

In addition to PDGF, several other RTK signaling pathways have recently been linked to the primary cilium, including fibroblast growth factor receptor (FGFR), epidermal growth factor receptor (EGFR) and insulin growth factor receptor (IGFR) signaling. A comprehensive review of these can be found in Christensen et al. (2012).

All of these RTK signaling cascades contribute directly or indirectly to regulation of mTor signaling, and conversely, mTOR signaling inhibits PDGFR alpha levels.

## mTOR signaling through the primary cilium

mTOR (mammalian target of rapamycin) pathway acts through mTOR complex 1 and 2 (mTORC1, mTORC2) and integrates information from a number of upstream pathways, including the tuberous sclerosis complex proteins such as tuberin. Polycystin-1 (PC1), the protein product of *PKD1*, mutated in the common ciliopathy autosomal dominant polycystic kidney disease (ADPKD) (Figure 7, Table 1), has been shown to interact with tuberin. The C-terminal cytoplasmic tail of PC1 interacts with tuberin, and plays a role in regulating mTOR. Patients with mutations in *PKD1* show inappropriate activation of mTOR in the epithelium of the kidneys (Shillingford et al., 2006). Rapamycin, an inhibitor of the mTOR pathway, can suppress cyst development in mouse models of polycystic kidney disease (PKD) and in human PKD patients after kidney transplant. Treatment with rapamycin can also induce apoptosis of cystic epithelial cells, reversing cystogenesis in PKD patients (Shillingford et al., 2006). Other studies in the Pkd1 mouse showed that mTOR hyperactivation was due to a failure of ubiquitination of c-met, a hepatocyte growth factor receptor (Qin et al., 2010). *In vitro* studies suggest that bending of the cilia caused by fluid flow results in downregulation of the mTOR pathway to control cell growth, possibly through Lkb1, a tumor suppressor protein localized to primary cilia (Boehlke et al., 2010).

More recently, OFD1, another cilia protein, has been implicated in functioning in the mTOR pathway. *Ofd1* mutant mice showed hyperactivation of mTOR in the kidney epithelia, which was successfully lowered with rapamycin treatment to significantly reduce cystogenesis (Zullo et al., 2010). Similarly, morphant zebrafish embryos deficient in various disease-causing ciliopathy genes found that treatment with rapamycin allowed significant rescue of normal phenotypes in most embryos (Tobin and Beales, 2008).

These findings, that the mTOR pathway functions through the primary cilium, and is inappropriately activated in kidney epithelia of PKD patients is of particular clinical significance, as cystic kidney disease is a common feature of multiple ciliopathies, and major cause of end-stage renal failure. Whilst the ciliopathies are individually rare, they are collectively common, particularly when considering PKD, and cystic kidney disease associated with ciliopathies represents a significant health burden. Research aimed at targeting the mTOR pathway to treat or prevent renal cytogenesis may lead to significant health benefits.

## GPCR signaling through the primary cilium

G-protein coupled receptors (GPCRs) (Dong et al., 2007) are essential for neuronal primary cilia function, and neuronal cilia integrity is essential for normal brain development and neuronal interactions in the adult brain. Various neurodevelopmental disorders occur when cilia function and structure are impaired, for example in the Joubert syndrome-related ciliopathies, schizophrenia and intellectual disabilities (Lee and Gleeson, 2011; Marley and von Zastrow, 2012). A recent whole genome siRNA knockdown screen for effectors of ciliogenesis found that neuroactive GPCRs were particularly enriched group of genes which, when knocked down, affect ciliogenesis. This highlights the importance of GPCRs for cilium structure and function, and identified many GPCRs previously not linked to cilia, which require further study (Wheway et al., 2015).

Neuronal cilium development begins with a pro-cilium (undifferentiated cilium lacking an axoneme) which forms after the neuronal cells have completed their migration and their mother centrioles dock to the cell membranes to form the basal body. The pro-cilium matures into a cilium postnatally over a period of 8–12 weeks. During mouse brain development migratory progenitor neurons from the ventricular zone differentiate into neural cells which are mostly directed to the upper layers of the neocortex. In the later stages of development, the presence of a pro-cilium coincides with the non-migrating neuronal population of the developing cortical plate (Arellano et al., 2012).

During post-natal development, the neuronal primary cilium membrane becomes equipped with GPCRs such as somatostatin receptor 3 (SSTR3) (Händel et al., 1999), melanin-concentrating hormone receptor 1 (MCHR1) (Berbari et al., 2008), serotonin receptor 6 (5HTR6) (Brailov et al., 2000), kisspeptin 1 receptor (KISS1R) (Koemeter-Cox et al., 2014), dopamine receptors 1,2, and 5 (D1, D2, and D5) (Marley and von Zastrow, 2010), neuropeptide Y receptors, NPY2R and NPY5R (Loktev and Jackson, 2013; Hilgendorf et al., 2016).

Different GPCRs localize to cilia membranes depending on the neuronal cell types. For example, KISS1R localizes specifically to the cilia of gonadotropin-releasing hormone neurons, and in absence of cilia there is a reduction of gonadotropin hormone release at the nerve terminals (Koemeter-Cox et al., 2014).

Sstr3 is targeted to neuronal cilia by Arl13b, critical for the interneuron connectivity and inhibitory circuit formation in the striatum of the mouse brain (Guo et al., 2017). Mutations in ARL13B are associated with classical Joubert syndrome in humans, associated with complex neurodevelopmental defects including cerebellar vermis hypoplasia, ataxia, and psychomotor delay (Cantagrel et al., 2008). The role of ARL13B in neuronal GPCR targeting to cilia is likely to contribute to the development of these phenotypes.

Overexpression of GPCRs such as SSTR3 or 5HT6 in the neocortex of the developing mouse brain causes premature and abnormal ciliogenesis presented by longer and branched cilia. This phenotype is associated with overexpression of IFT proteins such as Kif3a, cytoplasmic dynein D1, IFT88 and the GPCR ciliary trafficking protein TULP3. Furthermore, overexpression of 5HT6 and not SSTR3 prevent cilia localization of ACIII (Guadiana et al., 2013), a GPCR which is normally localized to the primary cilia of most neurons and is part of signal transduction cascades initiated by other receptors in the ciliary membrane (Berbari et al., 2007). Neuronal cells with abnormally long cilia or blocked cilia formation have abnormal dendrite outgrowth.

Conversely, other GPCRs have been shown to promote shortening of primary cilia. Recently, MCH was shown to induce primary cilia shortening in serum starved hTERT-RPE cells and was not affected by cell cycle control (Hamamoto et al., 2016). MCHR1 was involved in cilia shortening via the Gα_o−_Akt pathway but the precise mechanism of this process is still unclear. As the antennae of the cell, cilia length could influence the sensory potential of cells. Cilia shortening of the neurons of the hypothalamus was observed in high fat-diet induced obese mice (Han et al., 2014). Childhood obesity is a feature of Bardet-Biedl syndrome and Alström syndrome, multi-organ ciliopathies, and dysfunctional neuronal cilia could contribute to this phenotype (Mariman et al., 2016; Figure 7, Table 1).

Activated GPCRs are retrieved back to the cell through the scaffolding protein β-arrestin2 and the Bardet-Biedl Syndrome proteins in association with Arl6 (Bbs3/Arl6). When GPCR retrieval fails, for example in Arl6 knock-out cells, SSTR3 mutant cells or NPY2R mutants lacking motifs for recognition by BBSome and β-arrestin, the GPCRs concentrate at the ciliary tip and are subsequently ectocytosed alongside significant amounts of β-arrestin2 and the BBSome. Thus, neuronal cells with defects in cilia can have significant defects in GPCR signaling (Nager et al., 2017).

In addition to playing a role in the brain, GPCRs are also fundamentally important in the rod and cone photoreceptor cells of the retina, where the primary cilium has evolved to become highly specialized for the purpose of detecting light (Wheway et al., 2014). Rhodopsin in rods and opsins in cones are GPCRs which absorb light and transduce electrical signals via the optic nerve to the brain (Kiser et al., 2014).

As a result of the fundamental importance of the cilium in the retina, mutations in genes encoding proteins of the cilium often lead to disorders of retinal degeneration, either in non-syndromic Leber Congenital Amaurosis or retinitis pigmentosa or as part of syndromes such as Bardet-Biedl syndrome or Joubert syndrome (Figure 7, Table 1). Collectively these are termed the retinal ciliopathies (Bujakowska et al., 2017).

GPCRs are essential for normal neuronal cilia integrity and function. This includes in the outer segment of the photoreceptors of the retina. As a result, loss of cilia can lead to impaired GPCR signaling, and loss of GPCRs can lead to loss of cilia and/or cilia function.

## Cilia, signaling and human disease

Proteomics and functional genomics studies of ciliated cells and organisms suggest that between 1,200 and 1,800 genes are required for normal primary cilium structure and function in mammals (Gherman et al., 2006; Wheway et al., 2015). Mutations in many of these genes lead to defects in ciliogenesis, cilium structure or function. Due to their central role in cell signaling, loss of cilia, or abnormal cilia structure or function, results in significant signaling defects leading to diseases termed “ciliopathies.” The ciliopathies are a suite of conditions ranging in severity depending on the extent to which they affect ciliogenesis or cilium function, and therefore to what extent they affect signaling.

Throughout this review we have encountered ciliopathies resulting from specific signaling defects arising from loss or dysfunction of cilia. Table 1 summarizes these ciliopathy phenotypes and their underlying signaling defect resulting from cilia loss or dysfunction.

At the most severe end of the ciliopathy spectrum (Figure 7), Meckel-Gruber syndrome is a perinatal lethal condition affecting multiple organs including the kidneys, liver, central nervous system, lung and limbs. This syndrome can be caused by mutations in 15 different genes, most of which are located to the basal body or transition zone of the cilium and are essential for ciliogenesis. In this disease, null mutations in these genes lead to complete loss of proteins required for basal body migration, establishment of normal cell polarity and cilium growth. Absence of cilia on cells of multiple organs leads to severe developmental defects, with many features characteristic of the loss of key signaling pathways. Ciliopathies decrease in severity as the nature of the genetic mutation becomes less severe. In Meckel-Gruber syndrome, Joubert syndrome, Bardet-Biedl syndrome and nephronophthis many of the same genes are mutated, but the severity of resultant disease depends on how severely the mutation affects protein production or function. The less severe ciliopathies tend to be caused by hypomorphic mutations which do not prevent ciliogenesis entirely, but rather affect cilium function, sometimes in multiple organs, sometimes in specific organs. This determines the extent to which signaling is affected, and the severity of disease. At the mildest end of the ciliopathy severity spectrum, Leber congenital amaurosis and retinitis pigmentosa are conditions restricted to the retina, in which mutations selectively affect one specialized ciliated cell–the photoreceptor of the retina.

## Concluding remarks

Research over the past several decades has elucidated the broad and diverse ways in which primary cilia contribute to cell signaling. It has become clear that virtually all of the major signaling pathways in vertebrates converge on the primary cilium—it is truly the cell's antenna. It plays a particularly important role in Hedgehog signal transduction and is crucial for normal development. Future research may elucidate the confusion surrounding the role of primary cilia in canonical Wnt signal transduction, and perhaps show that the conflicts in the literature result from the context-dependent nature of Wnt signaling through the primary cilium. Although signaling pathways are often presented as linear, isolated pathways in the cell, in reality there is complex cross-talk between pathways, and constant dynamic fluctuations in signaling, dependent on time and space. Further to this, signaling varies according to cell type and genetic background, and study of the ciliopathies, conditions associated with extensive phenotypic heterogeneity, has aided our understanding of this. Study of the genetics of ciliopathies has taught us that phenotype in these conditions is significantly modified by genetic background (Khanna et al., 2009; Louie et al., 2010; Davis et al., 2011; Cardenas-Rodriguez et al., 2013), and study of ciliopathy animal models has offered insights into how this works at the level of signaling. For example, *Tmem67* mutant mice bred on different mice strain backgrounds were shown to develop Meckel-Gruber syndrome-like features or Joubert syndrome-like features, depending on their genetic backgrounds, and that at the molecular level this was associated with different defects in Hedgehog and Wnt signaling in the two conditions (Abdelhamed et al., 2013).

With the advent of systems biology approaches, we now have the ability to overlay multiple complex datasets and begin to understand the true complexity of signaling during human development, and its contribution to developmental disorders. The primary cilium represents an ideal model system for such an approach, being an organelle of reduced genetic complexity (estimated around 10% of genes encode proteins which contribute to functioning of the primary cilium). Recent whole genome sequencing studies, proteomics studies and reverse genomics studies are leading toward better understanding of the primary cilium and its function in human health and disease (Wheway et al., 2015; Boldt et al., 2016; Lindstrand et al., 2016; Shaheen et al., 2016).

Over the coming years, whole genome sequencing is likely to provide significant insight into the genetics underlying cilia function and dysfunction, as genomic testing becomes integrated into standard clinical healthcare in the UK National Health Service through the 100,000 Genomes Project. Around 50% of ciliopathy patients still do not receive a genetic diagnosis, even after genetic screening of all known ciliopathy genes, suggesting that much remains to be discovered about the genes and proteins important for cilium function and dysfunction. Population-scale whole genome sequencing studies, such as the 100,000 Genomes Project are enormously exciting opportunities both for patients to receive diagnoses, and researchers to gain insights into previously unknown pathways of disease. Widespread whole genome sequencing of ciliopathy patients will uncover mutations in novel ciliopathy genes, many of them likely to be private mutations, providing new understanding of the role of cilia in diverse cell signaling pathways, and potentially new targets for therapies. Perhaps the biggest challenge for researchers in this field is to develop techniques to identify which genetic variants from an estimated 50 million per individual are the variants likely to be significantly contributing to disease and warranting further investigation.

## Author contributions

GW was the main author and wrote the draft, while LN was a contributor, being a researcher in GW's laboratory. JH edited the paper and suggested some references to be cited.

### Conflict of interest statement

The authors declare that the research was conducted in the absence of any commercial or financial relationships that could be construed as a potential conflict of interest.
